# Axial forces and bending moments in the loaded rabbit tibia *in vivo*

**DOI:** 10.1186/1751-0147-54-21

**Published:** 2012-03-30

**Authors:** Janin Reifenrath, Daniel Gottschalk, Nina Angrisani, Silke Besdo, Andrea Meyer-Lindenberg

**Affiliations:** 1 Small Animal Clinic, University of Veterinary Medicine, Bünteweg 9, 30559 Hannover, Germany; 2 Institute of Continuum Mechanics, Appelstraße 11, 30167, Hannover, Germany; 3 Clinic for small animal surgery and reproduction, centre of clinical veterinary medicine, Faculty of Veterinary Medicine Ludwig-Maximilians-Universität München, Veterinärstr. 13, 80539, Munich, Germany

**Keywords:** Biomechanics, Hind leg, Telemetric, *in vivo*, Rabbit, Implant research, Tibia

## Abstract

**Background:**

Different animal models are used as fracture models in orthopaedic research prior to implant use in humans, although biomechanical forces can differ to a great extend between species due to variable anatomic conditions, particularly with regard to the gait. The rabbit is an often used fracture model, but biomechanical data are very rare. The objective of the present study was to measure axial forces, bending moments, and bending axis directly in the rabbit tibia *in vivo*. The following hypothesis was tested: Axial forces and bending moments in the mid-diaphysis of rabbit tibia differ from other experimental animals or indirectly calculated data.

**Methods:**

A minifixateur system with 4 force sensors was developed and attached to rabbit tibia (*n *= 4), which were subsequently ostectomised. Axial forces, bending moments and bending angles were calculated telemetrically during weight bearing in motion between 6 and 42 days post operation.

**Results:**

Highest single values were 201% body weight [% bw] for axial forces and 409% bw cm for bending moments. Whereas there was a continous decrease in axial forces over time after day 10 (*P *= 0.03 on day 15), a decrease in bending moments was inconsistent (*P *= 0.03 on day 27). High values for bending moments were frequently, but not consistently, associated with high values for axial forces.

**Conclusion:**

Axial forces in rabbit tibia exceeded axial forces in sheep, and differed from indirectly calculated data. The rabbit is an appropriate fracture model because axial loads and bending moments in rabbit tibia were more closely to human conditions than in sheep tibia as an animal model.

## Background

There are various animal models for musculoskeletal research. Some laboratory species, e.g. mice and rats, are too small for the investigation of implant materials (e.g. orthopaedic plates and screws) in weight-bearing bones. Therefore, in addition to sheep and dogs, rabbits are a commonly used model [1,2]. Studies using rabbit tibia were done to assess fracture healing following internal fixation with screws and plates [2,3], external fixation [4,5], and intramedullary nailing [6-8]. Although bone microstructure, bone remodelling, gait, and consequently, the biomechanical forces acting on the bones differ presumably from those of humans, screening of newly developed implant materials is common in animal models. As additional evaluation method, *in vivo *μ-computed tomography can be performed in rabbits to examine bone and implant alteration during the post operative follow-up period [9]. In contrast, this method cannot be used in larger animals due to their size. To decide, whether the rabbit or a large animal model is the appropriate choice for the aspired research question, knowledge of biomechanical forces is of utmost importance. Especially healing processes and possible implant failure have to be evaluated in dependency on the effective load to convey results to human conditions. Additionally, these data can be used to simulate bone remodelling [10] and to calculate the required stability of implant materials, even during the period of fracture healing, to avoid implant failure in advance.

For sheep, different force data already exists [11-13]. In comparison to calculated loads in human mid diaphysis of the tibia (maximum axial force 420% body weight [% bw]) [14], axial forces in sheep tibia were measured much lower (89% bw [11] and 110% bw [12]). These discrepancies might be explained with the quadruped gait. In contrast to sheep, the rabbit predominantly loads the hind legs during hopping and therewith approximate two-legged gait. However, there is a paucity of data regarding forces in the rabbit hind leg. An indirect method to calculate developing forces was described [15]. In that study, pins were surgically fixed in the femur, tibia, and metatarsus. Infrared light emitting diodes (LEDs) were attached to the pins, which were detected optically during hopping on a force measurement plate, enabling calculation of the corresponding forces. However, direct measurement methods using external fixateur systems are apparently only described for humans [16,17] and sheep [11-13], but these systems were not small enough to be adapted to the rabbit tibia. A telemetric method for axial force measurement in the rabbit tibia in free physiological movement has been reported [18]. The objective of the present study was telemetric evaluation of *in vivo *axial forces, bending moments, and bending moment angles in the rabbit tibia with weight bearing in free physiological movement to prove our hypothesis that axial forces and bending moments in the mid-diaphysis of rabbit tibia differ from other experimental animals or indirectly calculated data.

## Methods

**Figure 1:**
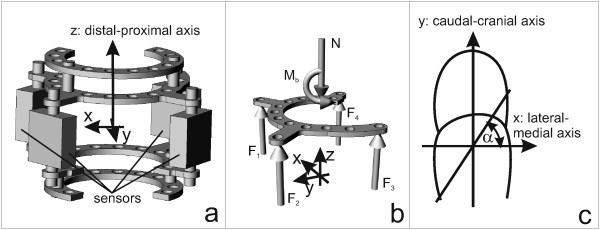
**Schematic diagram of the instrumented ring fixateur system**. Force sensors were placed between the middle 3/4-rings, enabling measurement of all axial forces between the proximal and distal sections of the ring fixator system (**a**); schematic diagram for calculation of the axial force N and bending moment M_b _(**b**). F_i _were forces measured in the force sensors; Calculation of the angle of the bending axis (α) to the lateral axis (0°) of the left hindleg (**c**); x, y, z mark the different axes.

Before *in vivo *measurements were performed, the system was tested *in vitro *as described for a similar system [18]. To measure axial forces and bending moments in the left tibia of New Zealand White rabbits, a ring fixateur system was designed based on a Smith & Nephew 3/4 ring fixation system (Figure 1a). The rings at the proximal and distal positions were commercially available 3/4 rings (50 mm in diameter; Fa Smith & Nephew, Marl, Germany). The two rings in the middle, specially designed to fix the force sensors, were aluminium to reduce mass. The two proximal and the two distal rings were consistently joined by two sleeves, threaded rods, and four screw nuts. The proximal and the distal parts were connected by four force sensors (KD24S, ± 100 N, Me-Meßsysteme GmbH, Hennigsdorf, Germany) (total weight approximately 250 g). The small s-shaped force sensors were oriented parallel to the bone and connected with a measurement amplifier via an associated board (GSV-4BT, Me-Meßsysteme GmbH, Hennigsdorf, Germany). The strain gauge measurement amplifier had a LiPo-battery, four ports, and wireless transmission. A standard Bluetooth dongle was used as a receiver and data were acquired, recorded, and processed using LabView^© ^(National instruments Germany GmbH, München, Germany).

Concurrent with force recordings, movement was recorded with a webcam, enabling calculation of the association between measured loadings and movement. Based on Newton's law [19] the axial force N was calculated as follows:

(1) $$N=F_{1}+F_{2}+F_{3}+F_{4}$$

where F_1,2,3,4 _were the forces measured with the four different force sensors (Figure 1b).

To calculate the bending moment M_b_, distances from each sensor to the medial-lateral axis and to the anterior-posterior axis were determined from radiographs. M_x_, the moment in the direction of the medial-lateral axis, was calculated as follows:

(2) $$M_{x}=-a\left(F_{1}+F_{2}\right)+b\left(F_{3}+F_{4}\right)$$

where a and b were the distances of the sensors to the lateral-medial axis. M_y _was the moment in the direction of the anterior-posterior axis and was calculated similar to M_x_.

(3) $$M_{y}=c\left(F_{1}+F_{4}\right)-d\left(F_{2}+F_{3}\right)$$

where c and d were distances between the sensors and the posterior-anterior axis.

The value of the maximum bending moment M_b _was calculated as follows:

(4) $$M_{b}=\sqrt{{M_{x}}^{2}+{M_{y}}^{2}}$$

The direction α of the bending moment was determined as:

(5) $$\alpha =\text{arctan}\frac{M_{y}}{M_{x}}$$

The angle was zero for the medial direction and it is mathematical positive defined (Figure 1c).

The experiment was conducted under a protocol approved by an ethics committee in accordance with German federal welfare legislation (AZ 509.6-42502/3-07/1304).

Adult female New Zealand White rabbits (n = 5, Charles River, Sulzfeld, Germany) were used. Anaesthesia was induced with S-ketamin hydrochloride (20 mg/kg, im) and medetomidin (25 mg/kg, im) and maintained with isoflurane (2-3 vol.%) in oxygen mixture (1 l/min 100% O_2_). After clipping and routine disinfection of the surgical field, the ring fixateur system was attached to the rabbit tibia. Holes were drilled in the bone and wires (1.0 mm in diameter; (Fa Smith & Nephew, Marl, Germany) were applied (tightened with 30 N tensile force). Two crossed wires were used in the middle half rings, whereas one wire was used for the proximal and the distal half ring to fix the threaded rods of the fixateur system parallel to the tibia. Before recovery from anaesthesia, radiographs were taken in four planes (0°, 45°, 90°, 315°) and distances between each force sensor and the central axis of the bone were measured.

**Figure 2:**
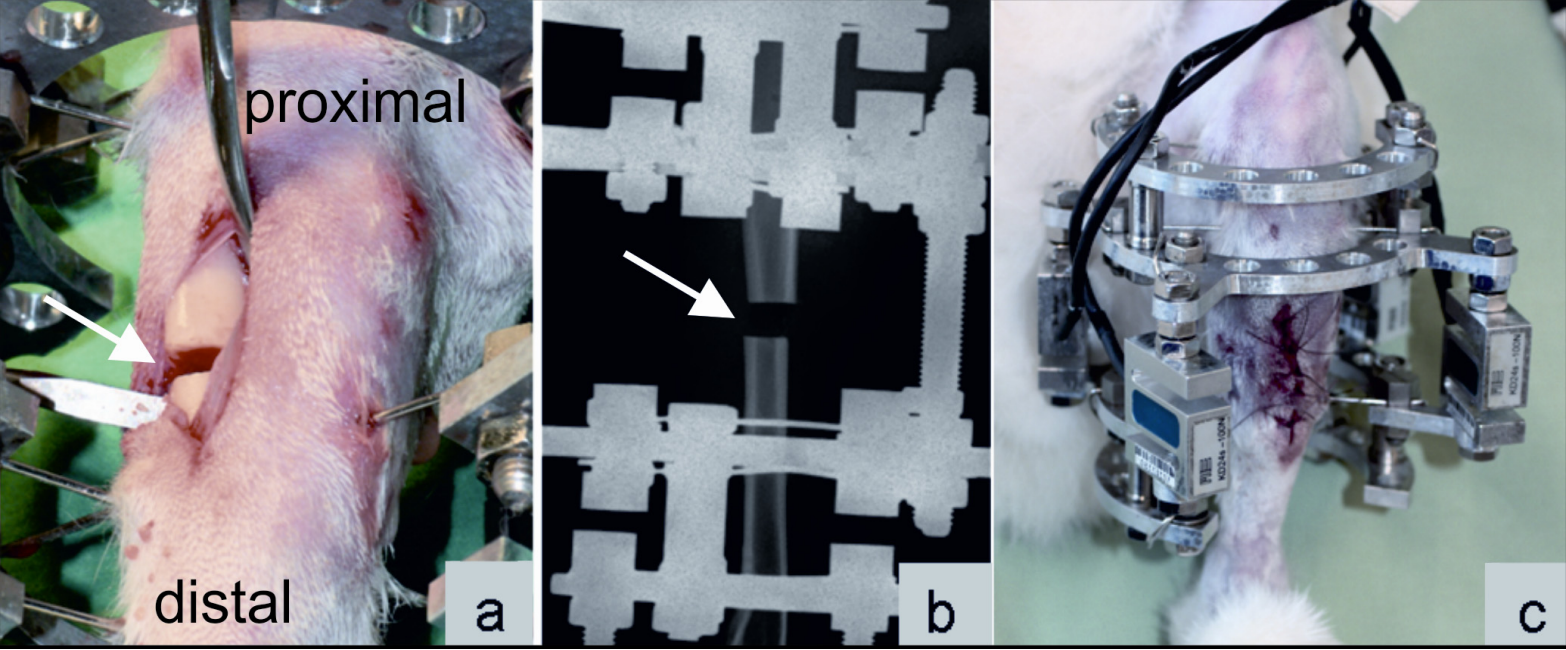
**Operative procedure of the rabbit tibia and preparation for measurement**. The rabbit tibia was ostectomised to transfer all developing forces via the force sensors: intraoperative picture (**a**) and post operative radiographic verification (**b**), ostectomy gap: white arrows; ring fixateur system with four pressure sensors after ostectomy of the tibia directly postoperatively for calibration of the force sensors under general anaesthesia without weight bearing (**c**)

After an adaption period of 2 weeks, four of the rabbits were anaesthetised and an ostectomy (3-4 mm) of the tibia was performed (Figure 2a). Following the ostectomy and radiographic verification (Figure 2b), the threaded rods were replaced by force sensors (Figure 2c). Consequently, all developing forces were transferred via the sensors [20]. The force sensors were connected (40 cm cables) to a board. Calibration of the force sensors without weight bearing was performed under general anaesthesia. Thereafter, the cables and board were securely wrapped in a bandage to protect them from damage between measurements.

In one rabbit, measurements without ostectomy were performed as control. The calibration procedure and measurement were done as described for the other rabbits. Three measurements were collected daily on 2 days. Mean maximum values and maximum single values of axial forces and bending moments were calculated.

Rabbits were given enrofloxacin (10 mg/kg, po) daily for 10d after surgery. A clinical examination was conducted daily to assess wound healing, swelling, pain, and lameness. For analgesia, meloxicam (0.15 mg/kg, po) was throughout the entire post operative measurement period, whereas buprenorphine (0.15 mg/animal) was given for 2d post operatively and prior to each measurement to prevent pain-induced reduced weight bearing.

Measurements with weight bearing in motion were conducted 6, 8, 10, 13, 15, 20, and 27d post operatively (three minutes per measurement, repeated three times each measurement day). In addition, one rabbit was measured at 34 and 41d post operatively. During measurements, the measurement amplifier and the LiPo-battery were fixed to the board of the force sensors and attached to a backpack to avoid adding an additional load to the leg. Data recordings were done as described above. During the measurement period, video recording was done to enable association of force and motion data. To calculate the average maximum bending moment for each measurement day, the 10 highest measured values were used. Student's t-test was used to detect differences between measurement days.

## Results

The ring fixateur system with installed force sensors was small enough to be attached to the rabbit tibia without causing clear visible changes in movement.

**Figure 3:**
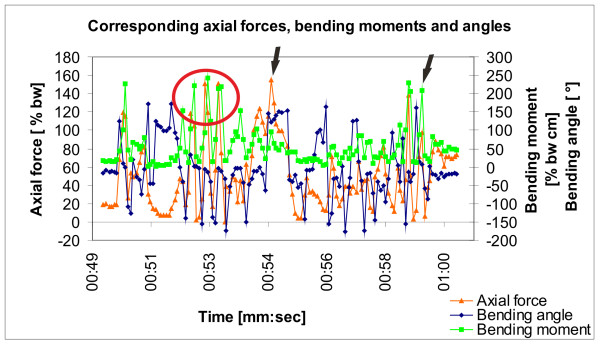
**Measurement of corresponding axial forces, bending moments, and bending angles in a rabbit tibia**. The corresponding axial forces, bending moments and bending angles during this measurement extract show that high values for bending moments were often associated with high values for axial forces (red circle) but this association was not consistent (black arrows). High bending moments usually occurred with a bending angle between 0 and -50°

Correlating axial forces, bending moments, and bending angles were be recorded during movement with weight bearing (Figure 3), and values were calculated in relation to body weight.

Maximum single values for axial forces were first measured 6 and 8 days post operatively in one and 3 rabbits, respectively (Table 1). During physiological movement, a maximum axial force of 201.41% bw as single value was recorded in Rabbit 4 (the most agile rabbit). Furthermore, an even higher value (233.68% bw) was recorded (during capture and restraint) of the same animal on the same measurement day. However, this value was excluded in the following calculations as it was not recorded during physiological movement.

**Table 1:** Maximum axial forces (single values) of each rabbit with the corresponding measurement day

Rabbit	Force [N]	Body weight [kg]	Force [% bw]	Measurement day
1	-60.32	3.98	154.54	6

2	-59.52	3.40	178.50	8

3	-54.64	3.89	143.24	8

4	-65.77	3.33	201.41	8

Control	-8.09	3.50	23.57	

**Figure 4:**
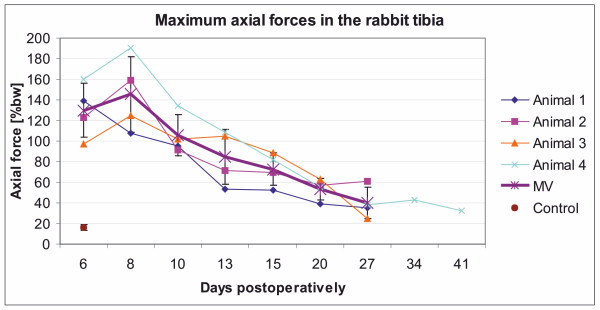
**The progress of developing maximum axial forces during the postoperative observation interval**. Measurements were performed until day 27 and in one rabbit additionally at day 34 and day 41, in comparison to a control animal without ostectomy (brown spot). A continous decrease in axial forces was apparent after day 8. The mean value of the ten maximum values of each measurement day were calculated for each animal (animal 1 to 4) and summarised for all animals (MV, stars). The ten maximum values were single measurement points, derived from the three measurement periods of the day.

Overall, there was an apparent increase in axial forces between days 6 and 8 (Figure 4), but it was not significant (*P *> 0.05). A continuous decrease in axial forces was apparent after day 10 (first significant at day 15, *P *= 0.03). Based on concurrent assessment of motion and data recording, maximum values for axial forces usually occurred at the beginning of hopping and sometimes during a hopping interval with changing speed.

**Figure 5:**
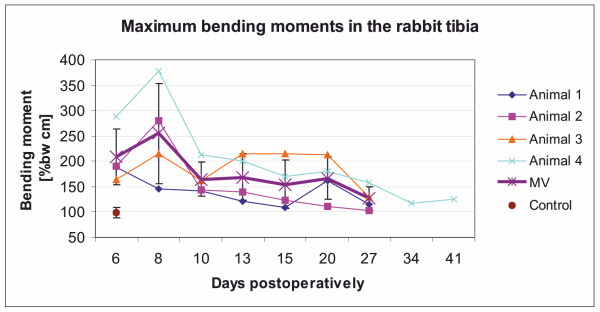
**The progress of developing maximum bending moments during the postoperative observation period**. Maximum bending moments of 27 and in one animal 41 days are shown in comparison to a control animal without ostectomy (brown spot). The mean value of the ten maximum values of each measurement day were calculated for each animal (animal 1 to 4) and summarised for all animals (MV, stars). The ten maximum values were single measurement points, extracted from the three measurement periods of the day.

Single maximum values for bending moments were documented at varying times in individual animals (Table 2) with no consistent decrease which could be observed in the values for axial forces (Figure 5). Significant differences between the time points could only be observed between day 6 and day 27 (*P *= 0.03). The highest value was documented in rabbit 4 (408.79% bw cm), which even had the highest values for axial forces. In rabbit 3, with 381.24% bw cm, only a moderate lower maximum value could be measured.

**Table 2:** Maximum bending moments (single values) of each rabbit with the corresponding measurement day

Rabbit	Bending moment [N cm]	Body weight [kg]	Bending moment [% bw cm]	Measurement day
1	80.110	3.98	205.25	6

2	127.11	3.40	381.24	8

3	105.18	3.89	275.72	15

4	133.50	3.33	408.80	8

Control	42.01	3.53	121.36	

Based on video analysis, maximum values for bending moments occurred at the start of hopping, in some cases during a hopping interval, and additionally when the animal changed the direction, for example in turns. Although, high bending moments corresponded with high axial forces, this did not occur consistently (Figure 3).

Bending angles occurred in all directions. In case of high bending moments, the corresponding bending angles were usually between -31° (25 percentile) and 3° (75 percentile), which represented a predominantly mediolateral direction.

**Figure 6:**
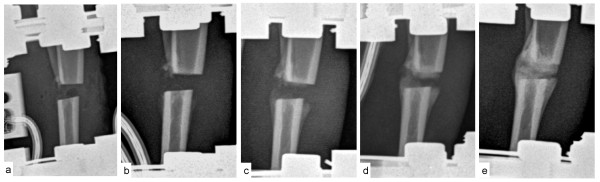
**Radiographic control of the ostectomy gap in Rabbit 4**. Callus formation was evaluated post operatively with radiographic pictures (medial-lateral view); the ostectomy gap is shown immediately post operatively (**a**), 13 (**b**), 20 (**c**), 27 (**d**) and 42 days post operatively. Radiopaque structures in the ostectomy gap were first detected 20 days post operatively and increased until day 42

Radiographic callus formation with radiopaque structures in the ostectomy gap were first observed 20d post operation with further evidence of healing during the post operative observation period (Figure 6).

## Discussion

For the use of fracture models in orthopaedic research, knowledge of developing forces is of utmost importance to compare mechanical loading with the situation in humans and to simulate bone remodelling [10] and the required stability of implant materials, even during the period of fracture healing.

Although the rabbit is a well-established animal model for orthopaedic research, apparently only indirect calculations of forces in the hind leg of this species have been reported [15]. Unfortunately, the rabbit hind leg is too small for direct methods of measurement as done in humans [16,17] and sheep [12,13]. In the present study, a new method for telemetric measurement of bending moments, bending angles and axial forces in the rabbit tibia with a very good clinical tolerance was developed.

The measured mean axial forces in the rabbit tibia with weight bearing in movement reached up to 201% bw. In previous studies, where axial forces without bending moments were measured [18], mean axial forces reached 152% bw. Furthermore, even higher axial forces (548% bw) were calculated for the tarsal joint of rabbits [15]. However these forces were indirectly calculated and additionally could be quite different from forces in the mid-diaphysis of the tibia, due to the effects of muscles and tendons. In comparison to calculated loads in human mid diaphysis of the tibia (maximum axial force 420% bw) [14] rabbits seem to reach more comparable loads in the mid diaphysis than sheep (measured axial forces of 89% bw [11] and 110% bw [12]). Perhaps the predominant load of the hind legs during hopping might have caused these discrepancies to the sheep.

The maximum bending moment in rabbit tibia in this study was 409% bw cm and thereby almost reached bending moments in human tibia (maximum bending moment in mid diaphysis up to 562%bw cm, calculated from the results of Wehner *et al. *[14]). In the rabbit hind leg, bending moments mainly occurred in a medial-lateral direction and much less often in an anterior-posterior direction. In a sheep model telemetrically measured bending moments in anterior-posterior direction were between 50 and 100 cm kilopond (which match 50 to 100 kg cm) for female sheep with a body weight of 50-55 kg [21]; this corresponded to an approximate value of 100-200% bw cm, but bending moments in medial-lateral were not considered [21]. Another theoretical calculation exists, where bending moments up to 39 Nm are evaluated during the stem phase [22]. With an assumed body weight of 50 kg, this value equates to 780% bw cm and therewith is much higher than the telemetric measured value in the *in vivo *study [21]. It is even higher than the measured values for the rabbit in our study. However a theoretical calculation can differ from measured results *in vivo*.

A decrease in axial forces and bending moments during the post operative observation period is caused by callus formation in the ostectomy gap. Consequently occurring forces only partially were transferred via the force sensors, depending on the callus stiffness. It is noteworthy that the axial force constantly decreased whereas no decrease in bending moments was detected. Fibrous tissue and beginning osseous callus formation in the ostectomy gap better bear up against axial forces than bending moments.

It is also noteworthy that allowing free movement, including changes in directions, provides a more comprehensive assessment of bending moments than measurements obtained with animals on treadmills, which are restricted to movement in only a single consistent direction. However, a possible influence of the mass of inertia of the system on the measurements during movements with acceleration cannot be excluded. In the moment of the maximum force during hopping, the system is accelerated in the opposite direction. Thus, the measured forces might be slightly lower than the real forces.

Nevertheless, our hypothesis was supported; axial forces in rabbit tibia exceeded axial forces in other experimental animals and differed from indirectly calculated data. For this reason, the rabbit is an appropriate animal model for fracture repair in orthopaedic research regarding the axial forces. Bending moments in rabbits even reached similar values than calculated data in human mid diaphysis of the tibia.

Thus, the rabbit is actually better qualified than the sheep because implants with adequate mechanical stability in sheep might fail in humans due to higher axial forces. A limitation for the use of rabbits is the animal size (evaluation of complex implant geometries is impossible) and a different histological bone structure [23], so that healing properties might be different.

## Conclusion

Compared to sheep, axial forces and bending moments in the rabbit tibia more approach axial forces and bending moments in humans. However, an entire comparison of bending moments between sheep, human and rabbit remains difficult due to incomplete directly measured data for bending moments in human and sheep. In conclusion, the rabbit is an appropriate animal model for fracture repair with simple implant geometries, especially due to its easy handling and the feasibility of additional examination methods like *in vivo *μ-computed tomography.

## Competing interests

The authors declare that they have no competing interests.

## Authors' contributions

JR carried out the study design, the animal measurements with data acquisition and drafted the manuscript. DG carried out the development of the measurement system and the calculation of data. NA participated in the animal measurements and data collection. SB participated in the design of the study, the measurement system and data calculation and helped to draft the manuscript. AML supervised the study, participated in its design and coordination and performed the operation procedure. All authors read and approved the final manuscript.
